# Clinical Feasibility and Safety of Nasal Allergen Provocation Testing in Pediatric Allergic Rhinitis

**DOI:** 10.1002/iid3.70487

**Published:** 2026-07-27

**Authors:** Mei‐Lan Wang, Terese Huiying Low, Zheng‐Cai Li, Xian‐Yun Bi, De‐Yun Wang, Jing Ma, Tie‐Song Zhang, Ying‐Qin Gao

**Affiliations:** ^1^ Department of Otolaryngology, Head and Neck Surgery Children's Hospital Affiliated to Kunming Medical University (Kunming Children's Hospital) Kunming China; ^2^ Department of Otolaryngology, Yong Loo Lin School of Medicine National University of Singapore Singapore Singapore; ^3^ Department of Otolaryngology, Head and Neck Surgery National University Health System Singapore Singapore

**Keywords:** active anterior rhinometry, allergic rhinitis, children, house dust mite, nasal allergen provocation testing, symptom scores

## Abstract

**Background:**

Nasal allergen provocation testing (NAPT) has been shown to be a safe and reproducible test in adults; however, pediatric data are limited. This study aims to investigate the clinical applications, feasibility, reproducibility and safety of NAPT in the pediatric population.

**Material and Methods:**

In this prospective study, 294 patients (4.16–14.25 years old, mean of 7.33 years), with symptoms and medical history of allergic rhinitis (AR) and IgE‐mediated sensitization to *Dermatophagoides pteronyssinus* and *Dermatophagoides farinae* were recruited. Participants underwent NAPT with negative control and with increasing concentrations of crude *Dermatophagoides farinae* (50 µg/ml, 500 µg/ml, and 5000 µg/ml) at 15 min intervals. Pre‐ and post‐test subjective symptom scores and objective active anterior rhinometry measurements were analyzed.

**Results:**

Among the 245 participants (83.3%) with a positive NAPT, the majority (*n* = 193, 78.8%) tested positive at 500 µg/ml or less. They (*n* = 226, 92.2%) were diagnosed based on an increase in total nasal symptoms score (TNSS) ≥ 5 points. The remaining 7.8% (*n* = 19) required objective measurements to fulfill the criteria for a positive result. Five participants were unable to fully comply with the instructions provided. There were no adverse events recorded during the study. The group which tested positive on NAPT recorded higher sIgE levels than that which tested negative. Furthermore, those with higher serum sIgE levels tested positive on NAPT at lower allergen concentrations. The baseline visual analog scale (VAS) was significantly higher in the group testing positive on NAPT at the lowest allergen concentration, compared with the other groups.

**Conclusion:**

Our study demonstrates that NAPT is safe and feasible to be conducted on the pediatric population.

## Summary

Nasal allergen provocation testing (NAPT) has been shown to be a safe and reproducible test in adults; however, pediatric data are limited. This prospective study evaluates the clinical application, feasibility, and safety of NAPT in children with allergic rhinitis. NAPT enables precise identification and confirmation of the causative allergen, especially in polysensitised individuals, making it a useful tool for planning and monitoring allergen immunotherapy.

## 1 Introduction

Allergic rhinitis (AR) is one of the most common chronic diseases, affecting approximately 500 million individuals worldwide. The disease often begins from early childhood, affecting up to 40% of children by 6 years of age [1]. A Meta‐analysis by Wang RK et al reported a prevalence of 18.46% in children aged 6 to 13 years‐old in China, between 2001 and 2021 [2, 3, 4]. The incidence of AR continues to rise, proving to be a major and increasing global healthcare burden, given its negative impact on quality of life and productivity [3, 5, 6]. This can manifest in children as fatigue, reduced attention span, poor memory and learning impairment, which may be overlooked or misinterpreted by their caregivers as having behavioral problems [4].

The diagnosis of AR is clinched based on history and supporting physical examination findings. Owing to the limited self‐reporting ability of younger children, physicians often rely on symptom reports by caregivers, of which both sources have been shown to affect the accuracy of diagnosis [7, 8]. Allergy testing is performed for patients with a clinical diagnosis of AR who do not respond to empiric treatment, when the diagnosis is uncertain, or when knowledge of the specific causative allergen is required for more targeted therapy [9].

The NAPT is an in‐vivo allergy test which involves direct and controlled exposure of nasal mucosa to the allergen, thereby inducing and measuring the allergen response. Its clinical applications are for the diagnosis of both allergic and non‐allergic rhinitis, and is a well‐established technique included in the standard diagnostic work‐up for rhinitis in some centers. It is also used to identify implicated allergens in polysensitised individuals, plan and monitor response to allergen‐specific immunotherapy [10, 11, 12].

NAPT has been proven to be a safe and reproducible test in adults, but there is limited data focused on the pediatric population [13]. There are at present no large‐scale studies nor standardized protocol for administration of the test in children [14, 15]. Therefore, this prospective study aims to present the author's experience with clinical NAPT in a cohort of 294 pediatric AR patients. It will also discuss the clinical applications, feasibility, reproducibility and safety of this test in the pediatric population.

## 2 Methodology

### 2.1 Study Population and Recruitment

All children diagnosed with AR induced by house dust mites, at the Department of Otorhinolaryngology (ENT) at Kunming Children's Hospital, between April to November 2023 were considered for the study. Each child was then assessed by two experienced ENT doctors to confirm their diagnosis. A total of 294 pediatric patients who met all the inclusion criteria and none of the exclusion criteria were enrolled (Table 1). Informed consent was obtained from the patient and legal guardian where appropriate.

**Table 1:** Estimated doses and PPV.

Positive predictive value	Dose estimate (㎍/ml)	95%CI lower limit (㎍/ml)	95%CI upper limit (㎍/ml)
0.010	1.585	0.067	5.858
0.020	2.624	0.164	8.502
0.030	3.614	0.287	10.808
0.040	4.597	0.437	12.980
0.050	5.592	0.613	15.094
0.060	6.606	0.817	17.192
0.070	7.645	1.049	19.297
0.080	8.713	1.311	21.429
0.090	9.814	1.603	23.601
**0.100**	**10.950**	**1.928**	**25.825**
0.150	17.232	4.069	38.056
0.200	24.708	7.199	53.020
0.250	33.659	11.472	72.134
0.300	44.431	17.027	97.378
0.350	57.467	23.982	131.641
0.400	73.357	32.456	179.208
0.450	92.901	42.610	246.551
**0.500**	**117.213**	**54.692**	**343.706**
0.550	147.887	69.099	486.780
0.600	187.287	86.453	702.732
0.650	239.074	107.725	1039.052
0.700	309.219	134.468	1584.926
0.750	408.173	169.294	2522.322
0.800	556.050	216.967	4267.082
0.850	797.299	287.350	7940.728
0.900	1254.709	405.516	17505.364
0.910	1399.928	440.186	21212.643
0.920	1576.798	481.013	26146.132
0.930	1797.174	530.043	32920.225
0.940	2079.902	590.424	42602.532
0.950	2457.033	667.327	57200.865
0.960	2988.369	770.008	80921.473
0.970	3801.539	917.278	124076.858
0.980	5234.889	1155.967	219301.242
0.990	8667.759	1660.181	539509.597

*Note:* Bold values indicate statistically significant.

Inclusion criteria:

- Aged between 4 and 18 years.
- Meeting the diagnostic criteria for allergic rhinitis, exhibiting at least two of the four classic symptoms (sneezing, nasal itching, runny nose, nasal congestion).
- Tested positive for allergies to Dermatophagoides pteronyssinus and/or Dermatophagoides farinae, based on positive skin prick test (SPT) and/or serum specific immunoglobulin E (sIgE).

Exclusion criteria:

- Previous or ongoing allergen immunotherapy treatment (AIT).
- Previous severe allergic reactions or anaphylaxis.
- Severe and uncontrolled bronchial asthma or chronic obstructive pulmonary disease.
- Severe comorbidities e.g. cardiopulmonary diseases.
- Other severe systemic diseases e.g. malignancy, autoimmune diseases.
- History of atrophic rhinitis, severe nasal congestion or severe epistaxis.
- Presence of nasal deformities or structural abnormalities affecting nasal airway patency e.g. choanal atresia, severe nasal septum deviation, nasal septal perforation, nasal polyps.
- Ongoing acute inflammation of the nose or paranasal sinuses (or within 4 weeks).
- Acute viral or bacterial infection (within 4 weeks).
- Recent vaccination (within 1 week).
- Recent sinonasal surgery (within 8 weeks).
- Study falling within the washout period for anti‐allergy medications.
- Within 2 weeks: nasal antihistamines, nasal decongestants, nasal anticholinergics, nasal cromolyn sodium, and oral antihistamines.
- Within 4 weeks: oral leukotriene receptor antagonists, nasal or injectable corticosteroids.

### 2.2 Skin Prick Test

Standardized allergen extracts for skin prick test (SPT, Wolwo Bio‐Pharmaceutical Co. Ltd., Zhejiang, China) were employed for the skin prick testing. Prior to commencement of the SPT, the cross‐scratch method was first used to mark out the prick positions on the volar aspect of the forearm, with adjacent markings spaced between 2 and 4 cm apart. The test area was then cleansed with 0.9% sodium chloride solution then left to rest for at least 2 min. Four pricks were administered to each participant, each containing separate standardized preparations of *Dermatophagoides pteronyssinus*, *Dermatophagoides farinae*, a negative control (0.9% sodium chloride solution) and positive control (10 g/L histamine phosphate solution).

A single drop of allergen solution was placed on the skin at the planned prick position, and a prick was made at the center of the droplet to allow the allergen solution to penetrate subcutaneously. If a strong skin reaction was observed 5 min before the remaining test solution present on the location of the administered prick was immediately wiped off. If no such strong reaction was observed, remnant allergen solution was wiped off after 5–10 min.

After 15 min, each skin wheal was measured, with a wheal diameter of ≥ 3 mm considered to be positive. Should the positive control elicit a negative reaction, the test was discontinued, and the patient excluded from the study. The Average Wheal Diameter (AWD) and Skin Response Index (SI) were calculated for each wheel, using the following formulas:

AWD=(maximumdiameter+perpendiculardiameter)/2.

SI=averagediameter/averagediameterofpositivecontrolwheel.

SPT results were graded from 1+ to 4+ based on SI: Grade 1+ for SI < 0.5 mm; Grade 2+ for SI ≥ 0.5 to < 1.0 mm; Grade 3+ for SI ≥ 1.0 to < 2.0 mm; Grade 4+ for SI ≥ 2.0 mm.

### 2.3 Serum Allergen‐Specific IgE Testing

4 mL of peripheral venous blood were collected from each patient. Centrifugation was performed for serum extraction. The UniCAP method allergen test kit (Phadia, Sweden) was used to detect serum sIgE antibody levels for each allergen. sIgE result was classified from levels 0 to 6 based on concentration of measured serum sIgE, with level 0 defined as a negative test result, while levels 1–6 are defined as positive. Level 0 for the absence of serum sIgE (kU/L); Level 1 for serum sIgE 0.35–0.70 kU/L; Level 2 for serum sIgE 0.71–3.50 kU/L; Level 3 for serum sIgE 3.51–17.50 kU/L; Level 4 for serum sIgE 17.51–50.00 kU/L; Level 5 for serum sIgE 50.01–100.00 kU/L; Level 6 for serum sIgE > 100.00 kU/L.

### 2.4 Symptom Scoring

Choice of tools used for measurement of pre and post‐provocation symptom scores were the TNSS grading each symptom of nasal itch, sneezing, rhinorrhea, and nasal obstruction on a Likert scale (0 = none, 1 = mild, 2 = moderate, or 3 = severe), and a simple (VAS) whereby subjects place a mark on a 100 mm line to indicate the severity of individual or overall symptoms on a scale from 0 (none) to 100 (severe) [13].

### 2.5 Preparation and Dilution of Nasal Provocation Solution

A glycerin‐free house dust mite allergen extract was prepared in‐house. The production process for the dust mite extract was as follows: One gram of purified *Dermatophagoides farinae* (Wolwo Bio‐Pharmaceutical Co. Ltd., Zhejiang, China) was frozen in liquid nitrogen, ground till fine, then observed under a microscope to ensure no intact body parts were visible. The ground allergen particles were defatted with acetone and dried overnight. The defatted and dried allergen particles were extracted in a phosphate‐buffered saline (PBS) solution at a mass‐to‐volume ratio of 1:10 for more than 72 h. The extract was centrifuged and filtered by passing the supernatant through filters with decreasing pore sizes (a 0.22 μm filter). The concentration of the allergen extract was measured using the Bicinchoninic Acid (BCA) protein concentration method, with a resultant protein concentration of 5021 μg/ml.

### 2.6 Determination of Nasal Provocation Solution Allergen Concentration (Pre‐Testing)

The original *Dermatophagoides farinae* extract solution at a concentration of 5000 μg/ml was further diluted in PBS to 5 different concentrations. A total of 6 different concentrations of allergen solution were prepared: 5000 μg/ml (original solution); 500 μg/ml (1/10 dilution); 50 μg/ml (1/100 dilution); 5 μg/ml (1/1000 dilution); 0.5 μg/ml (1/10000 dilution); 0.05 μg/ml (1/100000 dilution).

**Figure 1:**
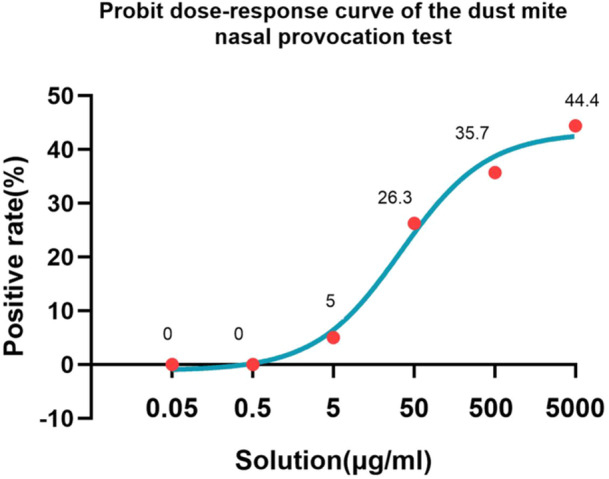
Testing of allergen concentrations on plot group. Red dots show the proportion of children with TNSS increase ≥ 5 points at the corresponding allergen concentration in the nasal provocation test.

Determination of appropriate concentrations of allergen solutions for NAPT was performed on a pilot group of 20 participants with *Dermatophagoides farinae* induced AR. Each participant was challenged at intervals with solutions of increasing allergen concentration, beginning with a concentration of 0.05 μg/ml, until a positive result was obtained or testing with a solution concentration of 5000 μg/ml was completed. A positive result is scored by an increase in TNSS score by ≥ 5 points compared to pre‐provocation baseline [11, 13]. The results showed that a positive reaction was observed in 1 participant (1/20, 5%) at a dose of 5 μg/ml, in 5 participants (5/19, 26.3%) at 50 μg/ml, in 5 participants(5/14, 35.7%) at 500 μg/ml, and in 4 participants (4/9, 44.4%) at 5000 μg/ml (Figure 1). Based on these results, with protein content as the “dose,” a probit regression analysis was performed to establish the dose‐response relationship. The following regression equation was obtained: Probit(P) = − 2.575 + 1.245 log_10_(dose).

The hypothesis test results for the regression equation yielded a *p* value of < 0.001, confirming the validity of the regression equation. The Goodness‐of‐Fit test was applied, with the following results: χ^2^ = 0.053, *p* = 0.997 > 0.05, indicating that the actual frequency and estimated frequency are consistent. Based on the above regression equation, the estimated dose values and Positive Predictive Value (PPV) were then calculated (Table 1). Based on the dose‐response relationship, the allergen concentrations determined for use in NAPT were 50 μg/ml, 500 μg/ml, and 5000 μg/ml. The recommended optimal dose is 500 μg/ml.

### 2.7 Nasal Allergen Provocation Testing

**Figure 2:**
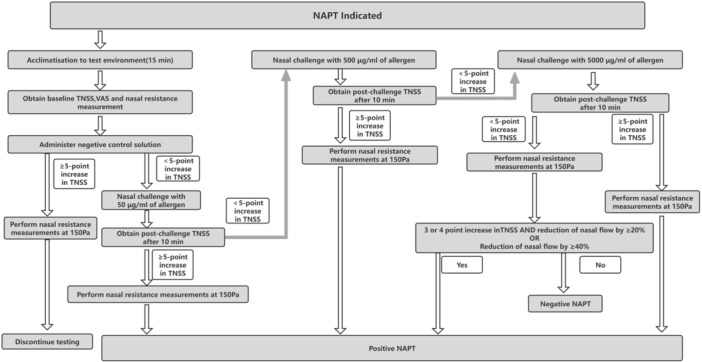
NAPT protocol.

Prior to commencement of the test, participants were required to spend 15 min acclimatizing to the test environment at an ambient temperature of 20 ± 1.5°C and humidity between 40% and 60%. Baseline patient demographic information, pre‐provocation nasal symptom scoring and nasal resistance measurements were obtained following acclimatization. The prepared concentrations of allergen and negative control (PBS) solutions were each held in identical 5 ml pump spray bottles, which were stored in a refrigerator at 4°C before and after testing. Prior to testing they were removed from storage and allowed to reach room temperature. Unused nasal provocation solutions were discarded 1 month from the date of manufacture. Prepared solutions were administered to the mucosa of the middle and inferior nasal turbinates via a spray method. Dose administered was standardized at 1 puff (100 μl/puff) of solution per nasal cavity. Where applicable, the prepared allergen solutions were administered in increasing concentrations till a positive NAPT result was obtained, or testing was completed using the solution containing 5000 μg/ml of allergen (Figure 2).

### 2.8 Nasal Resistance Measurement Technique

Nasal resistance was measured using active anterior rhinometry (Rhino31 Diagnostic Cube from ATMOS; Germany). All participants underwent nasal provocation testing using a stepwise increasing concentration protocol (50 µg/ml, 500 µg/ml, and 5000 µg/ml). After each step, the TNSS was assessed. If TNSS reached ≥ 5 points, dose escalation was stopped and nasal resistance was measured at that time; otherwise, provocation continued to the highest concentration.

Measurement technique was as per manufacturer's recommendations, with emphasis on the following steps: (a) The test environment is maintained at a temperature of 18°C–24°C and humidity of 30%‐75%. (b) The subject is seated, and refrains from any physical activity for at least 15 min before the examination. (c) The nasal pressure gauge is calibrated before use. (d) A suitably sized olive tip or nasal mask is selected based on the patient's nostril size, to ensure that a good seal is maintained during testing. (e) The subject is instructed to take several breaths, allowing them to adapt to the resistance of the olive tip and to establish a normal breathing pattern before the official measurements are made. (f) The subject is instructed to take 5 calm breaths; during which the computer automatically makes the relevant measurements and calculations, then records the examination results. If the breathing pattern is unstable or the young subjects do not cooperate well, the measurement is repeated. (g) After completion of the test on one side, it is then repeated on the contralateral nasal cavity. (h) Bilateral nasal resistance is then calculated from the measured right and left nasal resistance at 150 Pa, using this formula:

$$R\text{esistance}(R)=\frac{\text{Pressure Difference}\;(∆P)}{\text{Airflow}\;(V)}.$$

### 2.9 Scoring of NAPT Results

Subjective symptom score was obtained from the TNSS and VAS, while objective assessment of nasal resistance was conducted using active anterior rhinometry measurements. Post‐provocation scores were compared to baseline pre‐provocation scores.

The criteria for a positive NAPT result was as follows [11, 13]:

a. Increase in TNSS by 3 or 4 points AND nasal flow reduction of ≥ 20% at 150 Pa; OR
b. Increase in TNSS by ≥ 5 points alone; OR
c. Nasal flow reduction of ≥ 40% at 150 Pa

## 3 Results

Among the 294 participants, 4 participants developed a positive reaction to the negative control solution and did not proceed with formal NAPT. The remaining 290 participants successfully completed the study protocol.

### 3.1 Baseline Patient Characteristics

Of the participants who completed the NAPT, 182 were male (62.8%) and 108 were female (37.2%). The mean age of the participants was 7.33 years (range 4.16–14.25). Mean duration of symptoms was 2 years (range 0.02–10). 10 participants (3.5%) had comorbid asthma. 89 participants (30.7%) had a positive family history of atopy while 201 did not (69.3%).

All 290 participants tested positive to house dust mites on an allergy test other than NAPT. 215 participants (74.1%) tested positive to SPT only, 69 (23.8%) had positive serum sIgE test results while 6 participants (2.1%) tested positive to both SPT and serum sIgE testing. The mean overall quality of life score was 11. The mean baseline VAS rating was 4.

### 3.2 NAPT Results

A total of 245 participants (84.5%) tested positive, and 45 participants (15.5%) tested negative. In the positive group, 79 participants (27.2%) tested positive to the solution with an allergen concentration of 50 µg/ml, 114 (39.3%) tested positive to an allergen concentration of 500 µg/ml, and 52 (17.9%) tested positive to a concentration of 5000 µg/ml.

### 3.3 Subgroup Analysis of Baseline Characteristics

Patients were divided into four subgroups based on their NAPT result and concentration of allergen solution to which they tested positive (NAPT negative group, 50 µg/ml group, 500 µg/ml group, 5000 µg/ml group). Data followed a skewed distribution. Results were described using M (P25, P75). Subgroup analysis indicated no significant differences in duration of illness, age, gender ratio, or allergy status among the four groups (*p* > 0.05).

There were statistically significant differences in VAS scores and quality of life scores among the different challenge groups (*p* < 0.05). Further pairwise comparisons showed that the VAS scores of the negative group, 500 µg/ml group and 5000 µg/ml group were all lower than that of the 50 µg/ml group. There were no significant differences in VAS scores among the other groups (Table 2). The rhinitis quality of life questionnaire (RQLQ) score for the 50 µg/ml group was lower than that of the 500 µg/ml group, with no significant differences in RQLQ scores among the other groups (Table 2).

**Table 2:** Subgroup analysis on participants’ baseline characteristics [M(P_25_,P_75_)/*n*(%)].

Participant characteristics	NAPT negative (*n* = 45)	NAPT positive (*n* = 245)			*χ* ^2^ */H*	*P*
		50㎍/ml (*n* = 79)	500㎍/ml (*n* = 114)	5000㎍/ml (*n* = 52)		
Age (years)	7.58 (6.45, 9.70)	8.08 (5.75, 9.66)	7.20 (6.03, 9.85)	6.79 (5.58, 9.31)	1.807	0.613
Gender (Male/Female)	26/19	48/31	72/42	36/16	1.552	0.67
Duration of illness	2.00 (1.00, 3.00)	2.00 (1.00, 3.00)	2.00 (1.00, 3.00)	2.00 (1.00, 3.00)	3.957	0.138
Baseline VAS	4.00 (4.00, 5.00)	5.00 (4.00, 6.00)	4.00 (4.00, 5.00)	4.00 (4.00, 5.00)	15.278	**< 0.001** **
QoL score	10.00 (7.00, 13.00)	9.50 (7.00, 13.00)	12.00 (10.00, 13.00)	11.00 (7.00, 12.0)	10.466	**0.005** **
Allergy status					0.522	0.914
Single allergy [n(%)]	12 (26.7)	22 (27.8)	36 (31.6)	15 (28.8)		
Multiple allergies [n(%)]	33 (73.3)	57 (72.2)	78 (68.4)	37 (71.2)		

*Note:* Bold values indicate statistically significant.

* *p* < 0.05

** *p* < 0.01.

### 3.4 Changes in Post‐Provocation TNSS and Nasal Flow Values

Amongst those who successfully completed NAPT (*n* = 290), 226 participants had a post‐challenge increase in TNSS of ≥ 5 points, 37 participants had an increase of 3 or 4 points, and 27 participants had an increase < 3 points.

**Figure 3:**
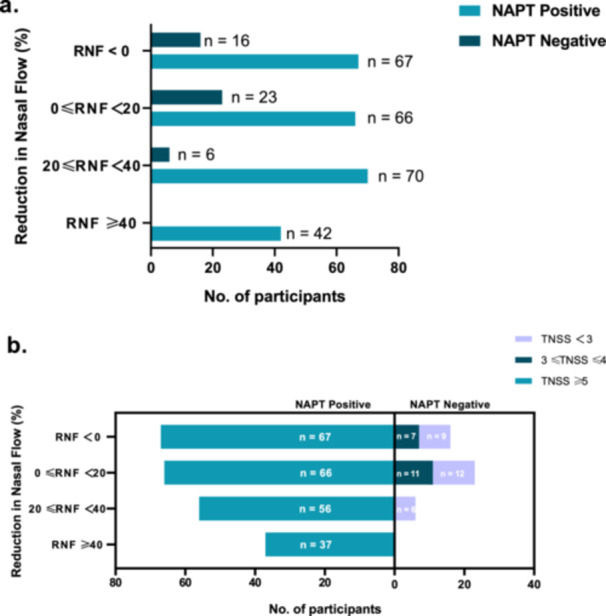
(a) Distribution of NAPT positive and NAPT negative patients across different nasal flow Changes. (b) The changes in TNSS and nasal flow in NAPT positive and negative patients. RNF, reduction in nasal flow.

In the group with positive NAPT results (*n* = 245), 226 participants (92.2%) recorded a post‐challenge increase in TNSS of ≥ 5 points. The remaining 19 participants (7.8%) recorded an increase of 3 or 4 points. In the group with negative NAPT results (*n* = 45), 27 participants (60%) recorded a post‐challenge increase in TNSS of < 3 points. The remaining 18 participants (40%) recorded an increase of 3 or 4 points. Figure 3 summarize and compare the changes in TNSS and nasal flow in NAPT positive and negative patients.

Five participants recorded a second nasal flow measurement that increased by more than double compared to the first. Parents of the 5 participants reported that their child was unable to cooperate during the measurements of nasal flow. 4 out of 5 of these participants were younger than 7 years of age (range 4.4–6.3 years). The parent of a participant, with a recorded 650% increase in nasal flow, reported that the child had difficulty complying with instructions and had blown their nose forcefully before the second measurement. No correlation was found between increase in TNSS and nasal flow reduction values in this group of patients.

### 3.5 NAPT, SPT and Serum sIgE Results

**Figure 4:**
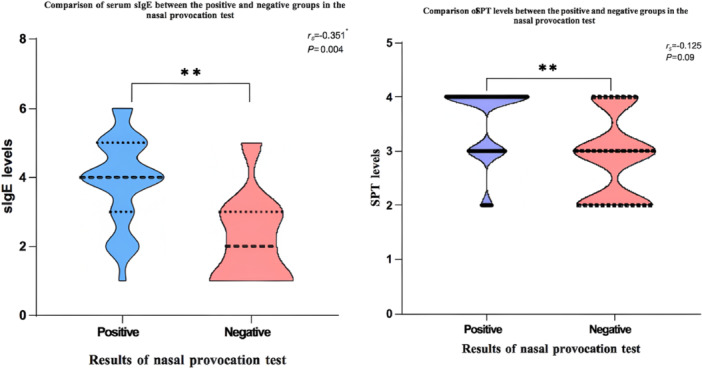
Comparison of SPT and serum slgE between the positive and negative provocation groups.

A statistically significant difference was detected in the SPT levels and serum sIgE levels between the positive and negative nasal provocation groups (*p* < 0.05). The positive group recorded higher serum sIgE levels compared to the negative group. There was no correlation between the nasal provocation solution concentration and the SPT grade (*p* > 0.05), while the nasal provocation solution concentration was found to have a negative correlation with serum sIgE levels (*p* < 0.05), with a correlation coefficient of rs = − 0.351 (Figure 4).

### 3.6 Individual Nasal Symptom Scores at Different Allergen Provocation Concentrations

Post‐provocation nasal symptom scores for each symptom of sneezing, rhinorrhea, nasal congestion and nasal itching were compared to the concentration of allergen at provocation.

Comparison of four nasal symptoms at three different provocation concentrations showed significant difference in symptom severity score between the positive and negative groups (*p* < 0.05). The positive group scored higher for all four symptoms. (Table 3). The cutoff value of 0.5 corresponds to the maximum Youden index, with the receiver operating characteristic curve (ROC curve) point closest to the upper left corner, representing the optimal combination of sensitivity and false positive rate.

**Table 3:** Comparison of four nasal symptoms at three different provocation concentrations.

Allergen concentration	Symptom	AUC	95% CI	Sensitivity	Specificity	Cut‐off value
*50 µg/ml*	Sneezing	0.905	(0.86,0.95)	0.896	0.869	0.500
	Rhinorrhea	0.874	(0.834,0.914)	1.000	0.583	0.500
	Nasal Congestion	0.875	(0.826,0.925)	0.883	0.83	0.500
	Nasal Itch	0.896	(0.858,0.933)	0.649	0.951	1.500
*500 µg/ml*	Sneezing	0.914	(0.873,0.956)	0.873	0.922	0.500
	Rhinorrhea	0.844	(0.792,0.897)	0.982	0.515	0.500
	Nasal Congestion	0.817	(0.757,0.876)	0.836	0.789	0.500
	Nasal Itch	0.887	(0.842,0.933)	0.791	0.893	1.500
*5000 µg/ml*	Sneezing	0.824	(0.732,0.915)	0.744	0.844	0.500
	Rhinorrhea	0.832	(0.755,0.909)	0.615	0.891	1.500
	Nasal Congestion	0.768	(0.677,0.858)	0.949	0.484	0.500
	Nasal Itch	0.794	(0.707,0.881)	0.949	0.531	0.500

## 4 Discussion

In this study, all 290 pediatric patients who underwent NAPT were able to complete the test. Their ages ranged from 4.16 to 14.25 years, with a mean of 7.33 years. 84.5% participants (*n* = 245) tested positive on the NAPT. The remaining 15.5% of participants who had a negative NAPT result possibly either had a false positive result on their initial allergy test, or an allergen concentration of 5000 µg/ml was not high enough to trigger in them a clinical nasal allergic response.

Of the 245 participants with a positive NAPT result, the majority (78.8%) tested positive at allergen concentrations of 500 µg/ml or less. The remaining 21.2% tested positive to the highest available test concentration of 5000 µg/ml. Subgroup analysis indicated no significant differences in duration of illness, age, gender ratio or allergy status. However, the baseline VAS was significantly higher in the group which tested positive at the lowest allergen concentration, compared with the other groups. This highlights that patients with more clinically severe AR are triggered by allergens present in lower concentrations than those with mild disease.

92.2% of participants who tested positive were diagnosed based on an increase in TNSS ≥ 5 points. The remaining 7.8% required objective measurements to fulfill the criteria of a positive result. Interestingly, in the group which tested negative, 40% reported a modest increase in TNSS of 3 to 4 points post‐procedure. However, they did not meet the criteria for a positive result when objective measures were considered. We initially followed the EAACI Position Paper on standardizing nasal allergen challenges [11], which defines non‐specific nasal hyperreactivity as a response to the negative control during NAPT exceeding 50% of the diagnostic positivity threshold. Using this definition (i.e., 50% of TNSS ≥ 5), we classified children with a TNSS increase of ≥ 2.5 after the negative control as having non‐specific hyperreactivity. However, in practice this yielded an unexpectedly high rate (18.4%, 54/294): 4 children (1.4%) had a TNSS increase ≥ 5 and 50 (17.0%) had a TNSS increase of 2.5– < 5. Among these 50 children, subsequent allergen provocation was positive at 50 μg/ml in 19 (38%), at 500 μg/ml in 18 (36%), and at 5000 μg/ml in 11 (22%); only 2 (4%) remained negative. This high rate is inconsistent with most published literature, which reports very low or even absent non‐specific hyperreactivity [16]. We therefore used the standard positive NAPT criterion (TNSS increase ≥ 5) as the threshold for defining non‐specific hyperreactivity, yielding a rate of only 1.4%, consistent with previous reports. This suggests that unlike in adult patients, patient‐reported change in symptom score alone may not be a reliable indicator of disease severity when reported by children or by proxy of the caregiver, and objective measures are necessary for symptom corroboration [7, 8, 17].

The group which tested positive on NAPT recorded higher serum sIgE levels than that which tested negative. Of those who tested positive, it was found that those with higher serum sIgE levels tested positive at lower allergen concentrations. This is consistent with the fact that a higher serum sIgE level is associated with a higher likelihood of a clinical allergy rather than sensitization.

### 4.1 Clinical Applications of NAPT

NAPT is a well‐established technique included in the standard diagnostic work‐up for rhinitis in some centers. Although it is time‐ and resource‐consuming, it is currently the only available test to directly confirm the nasal reactivity to allergens and can confirm the diagnosis in instances where it is in question. This may reduce the costs associated with the inadequate management of rhinitis patients [18].

Local allergic rhinitis (LAR) as a condition has gained awareness in recent years and identifies as a subset of AR. LAR is a confined nasal allergic response in the absence of systemic atopy (negative sIgE or SPT to allergens) that is characterized by a positive NAPT. It is a chronic upper airway condition with a natural evolution towards a severe disease phenotype with asthmatic symptoms, and a good response to Allergen Immunotherapy (AIT) [19]. Handan Duman et al.'s prospective study reports a prevalence of LAR of 25%, among children previously diagnosed as having non‐allergic rhinitis [15]. 40% of children previously diagnosed as having Non‐allergic rhinitis (NAR) based on negative SPT were reclassified as having LAR from a positive NAPT, in another study by Fausto Yoshio Matsumoto et al. [20] patients with LAR may present with symptoms which do not respond adequately to empirical or standard medical treatment. Tsilochristou et al estimates the prevalence of LAR in a subset of children with chronic, difficult‐to‐treat rhinitis and no sensitization to aeroallergens to be 29.2% [21]. LAR remains underdiagnosed in both the adult and pediatric populations [13, 14, 21]. There are no clinical characteristics capable of differentiating LAR from other forms of childhood rhinitis [14]. NAPT is considered the gold standard for diagnosis of LAR.

NAPT is shown to be highly reproducible and specific in individuals with multiple allergies [13]. It allows for more precise identification and confirmation of the offending allergen especially in polysensitised individuals, which is instrumental when planning to initiate AIT. It is also a useful tool for monitoring objective clinical response during AIT, by utilization of the concept of threshold testing [22].

### 4.2 Feasibility and Reproducibility of NAPT in the Pediatric Population

The European Academy of Allergy and Clinical Immunology (EAACI) recommends NAPT for children above the age of 5 years, as young children may not be able to accurately report their subjective symptoms nor cooperate with nasal resistance testing [13]. In this study, five participants had difficulty complying with instructions to the active anterior rhinomanometry (AAR), thus recording anomalous results despite having completed the test. Four of them were younger than 6.5 years of age. While age is not an absolute contraindication, the ability of the child to understand and comply with instructions needs to be considered before prescribing the test.

Subjective questionnaires chosen for our pediatric study population are the TNSS and VAS. These are short and easy to complete. Objective measurements were performed using AAR, which is a sensitive and specific method currently accepted as the international standard for objective nasal patency measurements [13, 23]. AAR is non‐invasive, but requires the participant to follow instructions to appropriately time the measurements with the breathing cycle. Hence, the child should be deemed to have the ability to follow such instructions in order to complete the NAPT. An alternative objective measure to consider in the younger pediatric population is acoustic rhinometry which results are not effort‐dependent and may overcome the challenge posed in testing younger patients who are unable to fully comply with instructions [13]. Younger participants should be accompanied by their caregiver, which would help ease and acclimatize them to the test environment, encouraging the completion of the test.

A prospective analysis by Eguiluz‐Gracia et al of repeated NAPTs performed at 1–2‐month intervals in patients with AR, LAR, or nonallergic rhinitis (NAR) and in healthy controls. The reproducibility and positive/negative predictive values of 3 consecutive NACs performed in 710 subjects were 97.32%, 100%, and 92.91%, respectively. There were no false positive results in patients with NAR and in healthy adult controls. These results show that NAPT is reasonably reproducible in adults. Such studies on the pediatric population remain lacking [13, 18].

### 4.3 Safety of NAPT

A retrospective evaluation of NAPT results of 11,499 subjects, including 518 children, reported only four local adverse events, all of which occurred in adults and none in children. These adverse events were confined to the upper airway and of mild to moderate severity, which included uvular edema and palatal pruritus. The events occurred within the first 10 min of an allergen challenge and lasted 15 to 30 min. 99.97% of tests were well tolerated [13, 18]. Likewise, there were no adverse events recorded in our cohort of 290 pediatric patients in our prospective study. NAPT is thus a safe technique in children, and monitoring for adverse events during and immediately after the procedure should be part of the test protocol.

### 4.4 Limitations and Future Studies

NAPT is a well‐established clinical test, in which reproducibility has been proven in adults but similar studies on the pediatric population are lacking. Future similar studies on different pediatric populations beyond this test center will be helpful in confirming the reproducibility of the test and study protocol. Studies detailing the specific challenges associated with administering the NAPT in the pediatric population, and techniques for overcoming them, can provide practical knowledge for ensuring reliable and accurate test results.

NAPT can be performed by a variety of methods, but the lack of a uniform technique for performing and recording the outcomes in the pediatric population represents a challenge for those considering introducing NAPT to their clinical practice. These include recommendations on allergen concentrations and dilutions, subjective and objective tools for measuring clinical response to allergens. This presents a limitation of this study, which does not adopt other objective measurements of nasal obstruction such as acoustic rhinometry, nor objective measurements of inflammation such as nasal eosinophillia or nitric oxide. Further studies are required to address these gaps before a standardized pediatric test protocol can be established.

## 5 Conclusion

NAPT is a direct method of allergy testing via end‐organ provocation, proven to be both safe and reproducible in adults. Based on our study, it is safe and feasible to be conducted on the pediatric population. Further large‐scale studies are required to standardize a protocol for NAPT in this group of patients, for it to be adopted more widely in clinical practice.

## Author Contributions

M.L. Wang, D.Y. Wang, T.S. Zhang, Jing Ma, and Y.Q. Gao designed the study. All authors undertook study‐related procedures. M.L. Wang, Z.C. Li, X.Y. Bi, and Y.Q. Wang collected the data. M.L. Wang, T.H. Low, D.Y. Wang, T.S. Zhang, Jing Ma, and Y.Q. Gao analyzed and interpreted the data. M.L. Wang, T.H. Low, and D.Y. Wang wrote the article, and all authors reviewed the article. All authors approved the final version.

## Ethics Statement

The study obtained ethics approval from the Ethics Committee of Kunming Children's Hospital. (Ethics Approval Number: 2023‐03‐113‐K01).

## Conflicts of Interest

The authors declare no conflicts of interest.

## Supporting information


Supporting File 1

Supporting File 2

## Data Availability

The data that support the findings of this study are available on request from the corresponding author. The data are not publicly available due to privacy or ethical restrictions. The data that support the findings of this study are available from the corresponding author upon reasonable request. All data generated or analyzed during this study are included in the published article.
